# Effects of theory of mind performance training on reducing bullying involvement in children and adolescents with high-functioning autism spectrum disorder

**DOI:** 10.1371/journal.pone.0191271

**Published:** 2018-01-17

**Authors:** Meng-Jung Liu, Le-Yin Ma, Wen-Jiun Chou, Yu-Min Chen, Tai-Ling Liu, Ray C. Hsiao, Huei-Fan Hu, Cheng-Fang Yen

**Affiliations:** 1 Department of Special Education, National Kaohsiung Normal University, Kaohsiung, Taiwan; 2 Department of Child and Adolescent Psychiatry, Chang Gung Memorial Hospital, Kaohsiung Medical Center and College of Medicine, Chang Gung University, Kaohsiung, Taiwan; 3 Department of Psychiatry, Kaohsiung Medical University Hospital, Kaohsiung, Taiwan; 4 Department of Psychiatry, School of Medicine and Graduate Institute of Medicine, College of Medicine, Kaohsiung Medical University, Kaohsiung, Taiwan; 5 Department of Psychiatry and Behavioral Sciences, University of Washington School of Medicine, Seattle, WA, United States of America; 6 Department of Psychiatry, Children’s Hospital, Seattle, WA, United States of America; 7 Department of Psychiatry, Tainan Municipal Hospital, Tainan, Taiwan; Universite de Bretagne Occidentale, FRANCE

## Abstract

Bullying involvement is prevalent among children and adolescents with autism spectrum disorder (ASD). This study examined the effects of theory of mind performance training (ToMPT) on reducing bullying involvement in children and adolescents with high-functioning ASD. Children and adolescents with high-functioning ASD completed ToMPT (n = 26) and social skills training (SST; n = 23) programs. Participants in both groups and their mothers rated the pretraining and posttraining bullying involvement of participants on the Chinese version of the School Bullying Experience Questionnaire. The paired *t* test was used to evaluate changes in bullying victimization and perpetration between the pretraining and posttraining assessments. Furthermore, the linear mixed-effect model was used to examine the difference in the training effect between the ToMPT and SST groups. The paired *t* test indicated that in the ToMPT group, the severities of both self-reported (*p* = .039) and mother-reported (*p* = .003) bullying victimization significantly decreased from the pretraining to posttraining assessments, whereas in the SST group, only self-reported bullying victimization significantly decreased (*p* = .027). The linear mixed-effect model indicated that compared with the SST program, the ToMPT program significantly reduced the severity of mother-reported bullying victimization (*p* = .041). The present study supports the effects of ToMPT on reducing mother-reported bullying victimization in children and adolescents with high-functioning ASD.

## Introduction

Bullying victimization is one of the most distressing experiences for children and adolescents, particularly when it occurs over a prolonged period [1]. Children and adolescents with autism spectrum disorder (ASD) are at a high risk of being bullied. A recent meta-analysis reported that the pooled prevalence estimate for school bullying victimization was 44% among 17 studies and that school-aged children and adolescents with ASD were at a higher risk of school bullying victimization than were those without ASD [2]. Studies have revealed that children and adolescents with ASD who experienced school bullying were more likely to have suicidal ideation or attempt suicide than were those with ASD who did not experience school bullying [3, 4]. Moreover, research has also revealed a high rate of bullying behaviors in children and adolescents with ASD [5, 6]. The National Survey of Children’s Health in the United States found that 44% of parents reported that their children with ASD have perpetrated bullying behaviors to others [5]. The National Longitudinal Transition Study 2 in the United States found that 14.8% and 8.9% of adolescents with ASD are bullying perpetrators and victim-perpetrators in preceding year, respectively [6]. Perpetrating aggressive behavior intended to harm or distress others is one of core definitions of bullying. However, it is not easy to determine whether children and adolescents with ASD perpetrate bullying intendedly because that they may have difficulties to explain their intention of perpetrating aggression behaviors in detail. Given that both bullying perpetration and victimization may further aggravate social difficulties for the youths with ASD, prevention and intervention programs are warranted for bullying involvement in children and adolescents with ASD [3–6].

Regarding the high risk of bullying victimization in children and adolescents with ASD, studies have proposed several possible etiologies, including communication problems [1, 7, 8]; fewer friendships [7–9]; stereotyped behavior and interests [10]; and aggressive behaviors [11]. The role of theory of mind (ToM) skills in bullying involvement among children and adolescents with ASD has drawn the attention of researchers [1, 8]. ToM performance is the ability to attribute mental states to oneself and others as well as to predict the behavior of others on the basis of their mental states [12]. Moreover, ToM performance is considered a crucial element in the capacity to decode and understand social cues and, consequently, in the development of adaptive social behavior [13]. Individuals with ASD have deficits in ToM performance [14]. Difficulties in ToM performance among individuals with ASD may increase the risk of bullying victimization in several ways. First, difficulties in ToM performance can impair social interactions, such as deficits in pragmatic abilities, lack of pretend play and embarrassment, and empathy in individuals with ASD [15, 16]. Second, difficulties in ToM performance may markedly affect the social relationships of individuals with ASD because emotional and behavioral responses depend on understanding the mental states of others. Third, these difficulties can result in low empathy and incorrect interpretation of the intention of others. Fourth, individuals with ASD find it difficult to identify bullying [8]; therefore, they may react to others’ attitudes and behaviors inadequately and the risk of being bullied increases consequently. Fifth, difficulties in ToM performance may limit the friendship of individuals with ASD with others and therefore reduce the possibility to receive protection and assistance from others when they are bullied. These possible influences of difficulties in ToM performance on increased bullying victimization indicate that only training in social skills may not be sufficient and thus enhancing ToM performance may be necessary for individuals with ASD to reduce their risk of bullying victimization.

In children and adolescents with high-functioning ASD, ToM abilities are more developed; however, adolescents with ASD still score significantly lower than those without ASD [17, 18]. A study reported that the ToM performance of children and adolescents with ASD can be enhanced through training [19]. Another study revealed that ToM performance is positively related to bullying behavior in preschool children [20] and elementary school children [21, 22]. However, whether ToM Performance Training (ToMPT) can reduce the severity of bullying victimization and perpetration in children and adolescents with high-functioning ASD has not been examined. This study analyzed the effects of the ToMPT program on reducing bullying involvement in the aforementioned population, compared with those in a Social Skills Training (SST) program. We hypothesized that compared with the SST program, the ToMPT program would significantly reduce bullying involvement in children and adolescents with high-functioning ASD.

## Methods

### Participants

The Institutional Review Board of Kaohsiung Medical University approved the study. The study participants were enrolled from the child psychiatry outpatient clinic of an affiliated teaching hospital of Kaohsiung Medical University in Taiwan. Taiwan’s National Health Insurance (NHI) program is a compulsory universal health insurance program. According to the medical referral system of the NHI, patients could visit any healthcare provider including the outpatient clinics of teaching hospitals in Taiwan without transference of general practitioners. Therefore, the children and adolescents of the child psychiatry outpatient clinic in the present study are representative of those of similar age in Taiwan. The participants were required to meet the following criteria for inclusion in the study: (1) age, 6–18 years; (2) having a diagnosis of ASD according to the fifth edition of the *Diagnostic and Statistical Manual of Mental Disorders* [23]; (3) full-scale intelligence quotient determined using the Chinese version of the Wechsler Intelligence Scale for Children, fourth edition [24], >80; and (4) having the ability to communicate verbally with others without any difficulty based on their mother’s observation and clinical observation.

A total of 56 children and adolescents with high-functioning ASD were randomly assigned to the ToMPT (n = 28) and SST (n = 28) groups. Two child psychiatrists confirmed the ASD diagnoses on the basis of a clinical interview and history provided by the mothers. In the ToMPT group, 26 participants and their mothers completed the training program and all assessments, and two participants dropped out for personal reasons. In the SST group, 23 participants and their mothers completed the training program, and two participants dropped out for personal reasons; three participants completed the training program, but their mothers did not complete the posttraining assessments. No difference was observed in sex (Fisher’s exact test, *p* > 0.05) and age (Mann–Whitney U test, *p* > 0.05) between training completers and noncompleters. All mothers rated their children’s severity of social communication deficits on the Chinese version of the Social Responsiveness Scale [25, 26] before the training programs. All children and adolescents and their mothers provided written informed consent. Mothers also provided written informed consent to agree their children participating into this study.

### Measures

The severities of school bullying victimization and perpetration in the participants in the ToMPT and SST programs were assessed before training commencement (pretraining) and at training completion (posttraining). The self-reported Chinese version of the School Bullying Experience Questionnaire (C-SBEQ) was used to evaluate participant experiences of bullying victimization and perpetration in the previous 1 month, with 16 items answered on a Likert 4-point scale (0, never; 1, just a little; 2, often; and 3, all the time) [27, 28]. Items 1–8 evaluate experiences of bullying victimization, namely social exclusion; being called a mean nickname; being spoken ill of; being beaten up; being forced to work; and having money, school supplies, and snacks taken away. Items 9–16 evaluate experiences of bullying perpetration. Higher total scores of items 1–8 and 9–16 indicate more severe bullying victimization and perpetration, respectively. The results of a study examining the psychometrics of the C-SBEQ have been described elsewhere and supported favorable reliability and validity of the C-SBEQ [28]. In the present study, both the participants and their mothers used the C-SBEQ to rate the severity of the bullying involvement of the participants in the previous month. Cronbach’s α coefficient of the subscales of self-reported bullying victimization, self-reported bullying perpetration, mother-reported bullying victimization, and mother-reported bullying perpetration in the present study was .80, .70, .83, and .71, respectively.

### Intervention

The ToMPT and SST programs were conducted in the form of group interventions at the frequency of one session per week. The participants in each program were divided into two groups according to their age (10–14 and 15–18 years). The ToMPT group had 16 and 10 participants aged at 10–14 and 15–18, respectively. The SST group had 12 and 11 participants aged at 10–14 and 15–18, respectively. The training scenarios were also altered to ensure the role-playing was developmentally appropriate for the two age groups. The programs in the respective groups were conducted by the same instructors.

The ToMPT and SST programs were led by a researcher who reviewed and discussed the teaching materials and procedures with the instructors before each weekly session to ensure instructor adherence to the goals of each session. The mentioned researcher was a certified special education teacher and has been teaching individuals with ASD for approximately 20 years. The ToMPT program instructor was a special education teacher who has experience of approximately 10 years in teaching individuals with ASD. The SST program instructor was a clinical psychologist who had completed a comprehensive training course for cognitive–behavioral therapy and has clinical experience of treating children and adolescents for 8 years.

The 10-session ToMPT program was developed for teaching emotion understanding and belief attribution on the basis of suggestions from Howlin et al. [19]. The topics in the emotion understanding session included recognizing facial expressions across genders and ages as well as identifying situation-, desire-, and belief-based emotions. The topics in the teaching belief attribution session included understanding the principle of seeing that leads to knowing, first- and second-order false belief, nonliteral language, white lies, and sarcasm. In the ToMPT program the instructor also used the situations of bullying as the examples to help awareness of emotion.

The 10-session SST program provided instruction on unwritten social rules for daily life, and the topics included appropriate dressing, eating in an appropriate manner, common social interaction courtesy, and guidelines for making friends. The teaching materials were adapted from Liu [29], who illustrated hidden social rules in daily life in Chinese society regarding dressing, eating and drinking, living, leisure, school life, and making friends. These hidden social rules must often be formally taught to individuals with ASD. In the SST program the instructor also used the situations of bullying as the examples to help knowing necessary social skills.

Four components are shared by the ToMPT and SST programs, namely social problem solving, impulse control, conversation rules, and frustration and stress control. However, the instructors of both groups illustrated the components in different ways, with a motivational and behavioral approach in the ToMPT program and behavioral approach in the SST program. Both programs applied cognitive–behavioral techniques to enable the participants to gain knowledge and practice target behaviors in training sessions. The target behaviors in each session were modeled, role played, and coached to promote acquisition and generalization. All training sessions were highly structured and involved scripts and activities.

### Procedures

The participants in both study groups were assessed using the C-SBEQ before and after the training. The research assistants read the questionnaire to obtain responses if the participants could not read. All mothers simultaneously completed the C-SBEQ to rate the severity of bullying involvement in the participants.

### Statistical analyses

The paired *t* test was used to evaluate changes in bullying victimization and perpetration on the C-SBEQ between the pretraining and posttraining assessments in the participants in the ToMPT and SST groups. Furthermore, the linear mixed-effect model was used to examine the difference in the training effect between the ToMPT and SST groups. In this model, group (0: SST and 1: ToMPT) was considered the between-subject factor, time (0: pretraining and 1: posttraining) the within-subject factor, and their interaction (group × time) the training effect. All statistical analyses were performed using SPSS 20.0 (SPSS Inc., Chicago, IL, USA). A *p* value of <0.05 was considered significant for all tests.

## Results

Sex, age, and the severity of ASD symptoms at the pretraining assessment were compared between the ToMPT and SST groups, as shown in Table 1. The results indicated no significant difference in the aforementioned factors between the two groups.

**Table 1:** Comparisons of sex, age, and the severity of ADS symptoms between the two participant groups.

	ToMPT (*n* = 26)	SST (*n* = 23)	χ^2^ or *t*	*p*
**Sex, %**				
**Girls**	4 (15.4)	2 (8.7)	.508	.476
**Boys**	22 (84.6)	21 (91.3)		
**Age (years), mean (SD)**	13.8 (2.7)	13.6 (1.6)	-.292	.772
**SRS scores, mean (SD)**	112.2 (23.3)	111.5 (32.6)	-.088	.930

SRS: Social Responsiveness Scale; ToMPT: theory of mind performance training; SST: social skills training

Table 2 shows the pretraining and posttraining severities of bullying involvement in the ToMPT and SST groups and the results of the paired *t* test regarding the changes. No significant differences in the pretraining severity of self-reported victimization (*t* = .029, *p* = .977), self-reported perpetration (*t* = -.422, *p* = .675), mother-reported victimization (*t* = -.861, *p* = .394), mother-reported perpetration (*t* = -.577, *p* = .566) of bullying were found between the ToMPT and SST groups. The results of paired *t* test regarding the changes indicated that in the ToMPT group, the severities of both self-reported (*p* = .039) and mother-reported (*p* = .003) bullying victimization significantly decreased from the pretraining to posttraining assessments. No significant change was observed in the severities of self- or mother-reported bullying perpetration. In the SST group, the severity of self-reported bullying victimization significantly decreased from the pretraining to posttraining assessments (*p* = .027). No significant change was observed in the severity of mother-reported bullying victimization and self- or mother-reported bullying perpetration.

**Table 2:** Changes in pretraining and posttraining severities of bullying involvement in the ToMPT and SST groups.

	ToMPT group				SST group			
	Pre- training Mean (SD)	Post- training Mean (SD)	paired-*t*	*p*	Pre- training Mean (SD)	Post- training Mean (SD)	paired-*t*	*p*
**Self-reported victimization of bullying**	3.6 (3.6)	2.4 (2.8)	2.182	.039	3.6 (4.1)	2.6 (3.2)	2.371	.027
**Self-reported perpetration of bullying**	2.3 (2.7)	1.5 (2.7)	1.864	.074	2.0 (2.4)	1.4 (2.2)	1.842	.079
**Mother-reported victimization of bullying**	8.0 (4.6)	5.3 (3.0)	3.297	.003	6.9 (4.2)	6.4 (3.6)	.754	.459
**Mother-reported perpetration of bullying**	3.8 (3.4)	3.2 (2.9)	1.280	.212	3.3 (3.3)	3.5 (3.2)	-.646	.525

SD: standard deviation; SST: social skills training; ToMPT: theory of mind performance training

**Fig 1:**
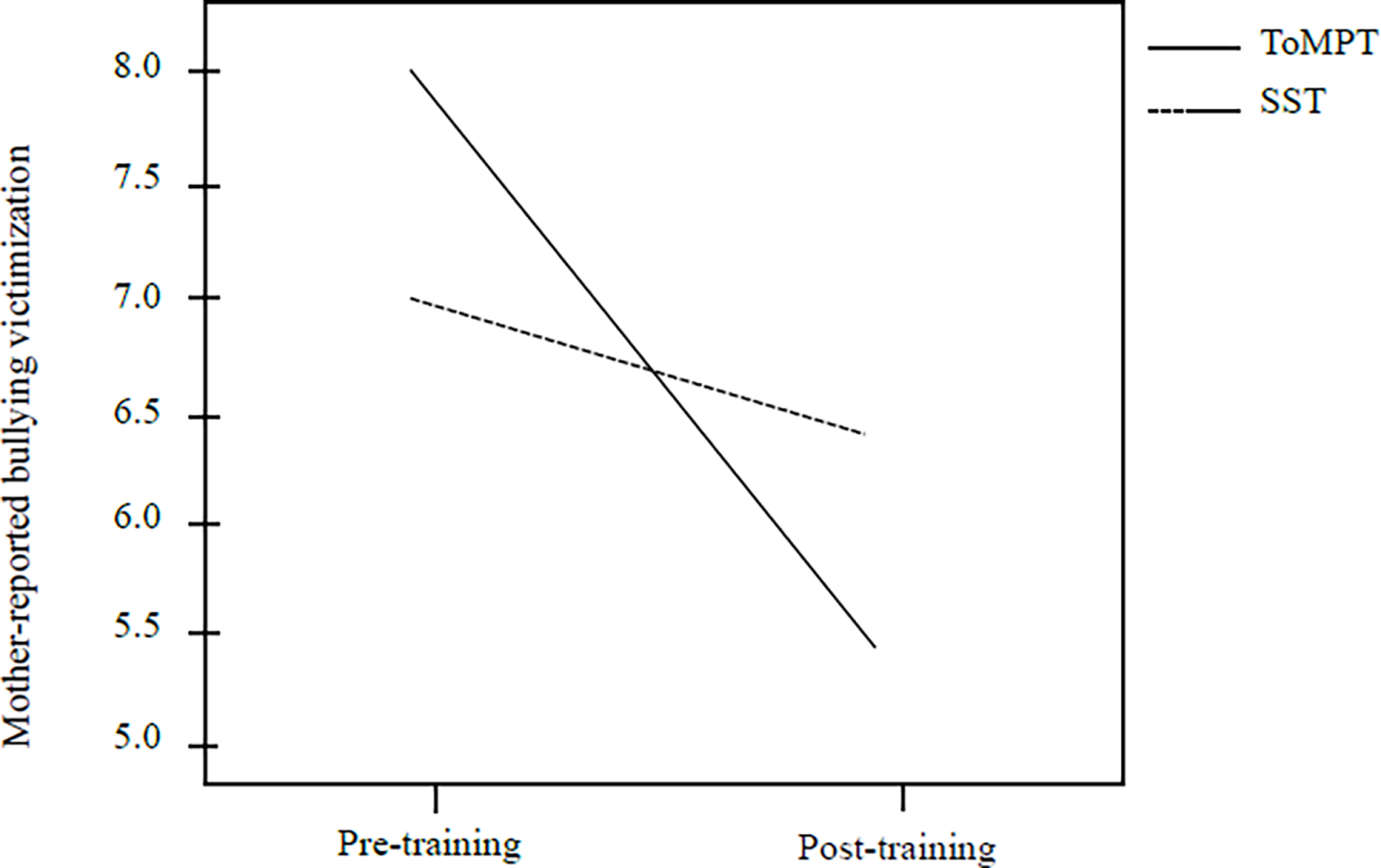
Changes in the severity of mother-reported bullying victimization from the pretraining to posttraining assessments in the study groups.

Table 3 shows the results of the linear mixed-effect model that examined the effects of training programs on self- and mother-reported bullying victimization and perpetration. The results indicated that compared with the SST program, the ToMPT program significantly reduced the severity of mother-reported bullying victimization (*p* = .041). Fig 1 shows the changes in the severity of mother-reported bullying victimization from the pretraining to posttraining assessments in both study groups.

**Table 3:** Effect of training programs on self- and mother-reported bullying victimization and perpetration, as examined using the linear mixed-effect model.

	Within-subject analysis					Between-subject analysis			
	df	Mean square	F	p		df	Mean square	F	p
Self-reported victimization of bullying									
Time	1	29.328	9.706	.003	Intercept	1	905.093	43.971	< .001
Group x Time	1	.226	.075	.786	Group	1	.399	.019	.890
Error	47	3.022			Error	47	20.584		
Self-reported perpetration of bullying									
Time	1	11.502	6.371	.015	Intercept	1	320.073	29.448	< .001
Group x Time	1	.359	.199	.658	Group	1	.849	.078	.781
Error	47	1.805			Error	47	10.869		
Mother-reported victimization of bullying									
Time	1	61.341	9.048	.004	Intercept	1	4335.602	184.181	< .001
Group x Time	1	29.912	4.412	.041	Group	1	.010	.000	.984
Error	47	6.780			Error	47	23.540		
Mother-reported perpetration of bullying									
Time	1	.767	.310	.580	Intercept	1	1159.146	65.484	< .001
Group x Time	1	4.685	1.894	.175	Group	1	.288	.016	.899
Error	47	2.474			Error	47	17.701		

Time: pretraining vs. posttraining; Group: theory of mind performance training vs. social skills training

## Discussion

The present intervention study revealed that the severity of self-reported bullying victimization significantly decreased from the pretraining to posttraining assessments in both the ToMPT and SST groups, indicating that both programs have positive effects on self-reported bullying victimization in children and adolescents with high-functioning ASD. However, the ToMPT program showed a superior effect on reducing the severity of mother-reported bullying victimization in the children and adolescents with high-functioning ASD than did the SST program. Additional studies are warranted to replicate the present findings to support the value of ToMPT for preventing and reducing bullying victimization in children and adolescents with high-functioning ASD.

Although the present study did not examine the exact mechanisms through which the ToMPT program reduces mother-reported bullying victimization in children and adolescents with high-functioning ASD, gaining an understanding of the possible mechanisms can facilitate revising the ToMPT program for children and adolescents with ASD. First, given that ToM performance involves the ability of participants to simultaneously consider their own and others’ mental states [15, 16], it is reasonable to hypothesize that ToMPT may reduce bullying victimization by enhancing the communication ability in children and adolescents with high-functioning ASD. However, a previous study did not reveal discernible improvement in conversational ability and the use of mental state terms in speech following ToMPT [30]. It is possible that conversation disability is one of core deficits of ASD and could not be significantly improved during ToMPT that targets on enhancing ToM performance. Thus, the result of the previous study [30] did not support the hypothesis that ToMPT may reduce bullying victimization by enhancing the communication. Second, ToMPT may improve the capacity of children and adolescents with high-functioning ASD to decode and understand social cues and consequently develop adaptive social behaviors, for example, predicting the behavior of others and maintaining distance from those who may bully them. Third, ToMPT may improve the capacity of children and adolescents with high-functioning ASD to realize that their own and others’ mental states can vary on receiving new information; thus, they can gain the experience of bullying victimization and develop alternative social interaction patterns. However, these proposed possible mechanisms warrant additional studies.

The present study reveals that the ToMPT program had a superior effect on reducing the severity of mother-reported but not self-reported bullying victimization compared with the SST program. We examined the severity of pretraining bullying victimization and revealed that the severity of mother-reported bullying victimization was significantly higher than that of self-reported bullying victimization in both the ToMPT (8.0 vs. 3.6) and SST groups (6.9 vs. 3.6). Studies have reported that the teacher-reported rate of bullying victimization in adolescents with ASD was higher than the self-reported rate [8, 31]. Researchers have supposed that because of the deficits in social insights, adolescents with ASD may have lower ability to recognize bullying than adolescents without ASD [8]. The present findings support that multiple information sources are required to delineate accurately the conditions of bullying involvement in children and adolescents with ASD. Moreover, compared with SST, ToMPT may have better efficacy to enhance child-mother interaction and thus mothers could help children and adolescents with managing bullying experience. However, the hypothesis warrants further examination.

Research revealed that adolescents with ASD have limited insights in social processes [32]; they may not be aware of the consequences of their own behavior and thus may bully, without being aware of it [8]. However, the present study determined that neither the ToMPT nor SST program reduced the severities of self- and mother-reported perpetration of bullying in the children and adolescents with ASD. The severity of bullying perpetration in the present study was low, thus limiting the possibility of changes in pretraining and posttraining assessments. In addition, children and adolescents may perpetrate bullying for various reasons, such as for demonstrating their physical and social superiority over others and for exploring their value and self-identity. Thus, the enhancement of emotion understanding and belief attribution by ToMPT might be inadequate to reduce bullying perpetration in children and adolescents with high-functioning ASD.

The present study is one of the first studies to analyze the effects of the ToMPT program on bullying involvement in children and adolescents with ASD. However, several limitations of this study must be addressed. First, the present study did not examine the levels of ToM performance and social skills and thus could not determine whether the ToM intervention improved ToM performance and whether the SST improved social skills. Second, the small sample size of the ToMPT and SST groups limited the possibility of examining the moderating effects of age, sex, and deficits in social cognition on the effect of the intervention programs. The influences of comorbid psychiatric diagnoses such as attention-deficit/hyperactivity disorder and anxiety and depressive disorder on the effects of intervention were not measured in the present study. Third, this study did not involve follow-up and could not calculate how long the effects of ToMPT persisted. Fourth, we did not use the gold standard diagnostic tool to confirm the diagnosis of ASD. Fifth, the reliability of the C-SBEQ in children and adolescents with ASD and their parents warrants further study. Moreover, no information regarding bullying involvement was obtained from the teachers and peers of the participants. Owing to the deficits of social cognition, children and adolescents with ASD may be not aware of bullying victimization or perpetration occurred. Parents may have difficulties in knowing their children’s experiences of bullying involvement and in interpreting whether the conflicts between the children and their peers conform to the characteristics of bullying. Information regarding bullying involvement from the teachers and peers permits the aggregation of judgment about individuals’ roles in bullying.

In conclusion, this study reports that compared with the SST program, the ToMPT program significantly reduced the severity of mother-reported bullying victimization in children and adolescents with high-functioning ASD. Because of the high rates of children and adolescents with ASD reporting being bullied and bullying-related mental health problems, the present findings may provide a basis for developing prevention and intervention programs for bullying victimization in children and adolescents with ASD.

## Supporting information

S1 Database SPSS database of qualitative responses to survey questions. (SAV)
